# Case Report: Virtual natural environment solution helped a child cope with a painful procedure

**DOI:** 10.3389/fped.2024.1355046

**Published:** 2024-05-02

**Authors:** Elina Karppa, Kaija Puura, Ilmari Jyskä, Markku Turunen, Sauli Palmu

**Affiliations:** ^1^ Tampere Center for Child, Adolescent and Maternal Health Research, Faculty of Medicine and Health Technology, Tampere University, Tampere, Finland; ^2^ Department of Children and Adolescents, Tampere University Hospital, Tampere, Finland; ^3^ Ostrobothnia Wellbeing Services County, Kuusamo Health Center, Kuusamo, Finland; ^4^ TAUCHI Research Center, Faculty of Information Technology and Communication Sciences, Tampere University, Tampere, Finland

**Keywords:** virtual reality, deep breathing, trypanophobia, analgesia, child

## Abstract

Fear of needles is a common phenomenon that can affect the patient's ability to function and to seek medical help. Novel treatment practices are needed to help children cope with this fear. Based on user feedback, immersive virtual reality applications are effective when distracting the patient during a painful procedure. Better understanding of how virtual reality solutions affect the autonomic nervous system should be acquired. We present the case of a 12-year-old boy attending our study examining a novel virtual reality (VR) relaxation method (VirNE). The clinical study aims to determine if pain and anxiety can be relieved by relaxation that has been induced by a virtual natural environment and guided relaxation exercise-mediated autonomic nervous system stimulation. The patient was able to overcome his fear of needles with the help of the guided relaxation and found significant relief from the distress he was experiencing on his monthly visits to the hospital due to his long-term illness requiring repetitious intravenous cannulations.

## 1 Introduction

Fear of needles is a relatively common phenomenon among children and youths (1). Approximately 2/3 of children are scared of punctures and injections at some level (2). Some of the patients experience severe anxiety before and during the procedure, affecting their ability to function (3). This phobia can lead to avoidance behavior and limitations on receiving health care services. The problem typically begins during early childhood and can extend to adulthood as trypanophobia, with a total life-time prevalence of 3%–4.5% (1). The autonomic nervous system is responsible for modulating the fight or flight response. When the patient is anxious or scared, the sympathetic nervous system is dominantly activated, elevating the heart rate and narrowing heart rate variability (4, 5). The patient can regulate the sympathetic nervous system-mediated fight response by volitional breathing and relaxation, which can be used as a tool of anxiety control.

Traditionally, the fear of needles has been treated by distraction or educating the patient about the up-coming situation (6). Topical numbing cream or sedative medication can alleviate the discomfort, but these methods are not always sufficient and do not facilitate the patients' capacity to self-regulate the autonomic nervous system response. Furthermore, sometimes child patients have been forced to be pricked with a needle despite protests or fright, creating a traumatizing event (7).

We are teaching children to relax during a painful procedure and cope with the unpleasant emotions they are experiencing by using a virtual reality relaxation application designed for pediatrics (VirNE) (8, 9). The children use virtual reality headsets and view a Finnish 360-degree virtual natural landscape as they perform a short, guided relaxation or deep breathing exercise during an intravenous cannulation. The restorative psychological effect of spending time in green environments has been demonstrated (10). A similar effect can be achieved by using virtual nature landscapes (11) or taking a short virtual forest walk (12), allowing the patient to experience the recreational effects of the outdoors irrespective of location, time, and functional ability or disability.

One of the benefits of utilizing virtual reality is a phenomenon called immersion, which refers to the highly engaging and realistic nature of being deeply involved in the virtual environments. Consequently, virtual reality solutions are capable of producing lifelike experiences, simulating one being present in the artificial surroundings, hence presumably affecting the nervous system response of the virtual reality user.

## 2 Case description

A 12-year-old boy was diagnosed with juvenile idiopathic arthritis (JIA) in 2017. Treatment with a biological sub-cutaneous medication, adamimulab (© Humira), was started in Tampere University Hospital Department for Pediatrics in May 2018. The medication was later switched to another biological drug, tocilitsumab (© RoActtemra), in December 2018. For the first 6 months the patient received the medication twice a month subcutaneously. Since June 2019, the drug was given once a month, but the less frequent dosing required intravenous administration at the site.

Intravenous cannulation was technically difficult, and the patient had a severe fear of needles. Nitrous oxide was given to ease his pain and anxiety, but it caused intense nausea as an adverse effect. From 2020, only an experienced anesthesiologist was able to accomplish the cannulation due to the technical challenges that resulted from the fear the patient was experiencing.

## 3 Therapeutic intervention and outcomes

In 2022, the patient participated in our study. The aim of the study is to find out if pain and anxiety can be relieved by the VirNE-application.

**Figure 1:**
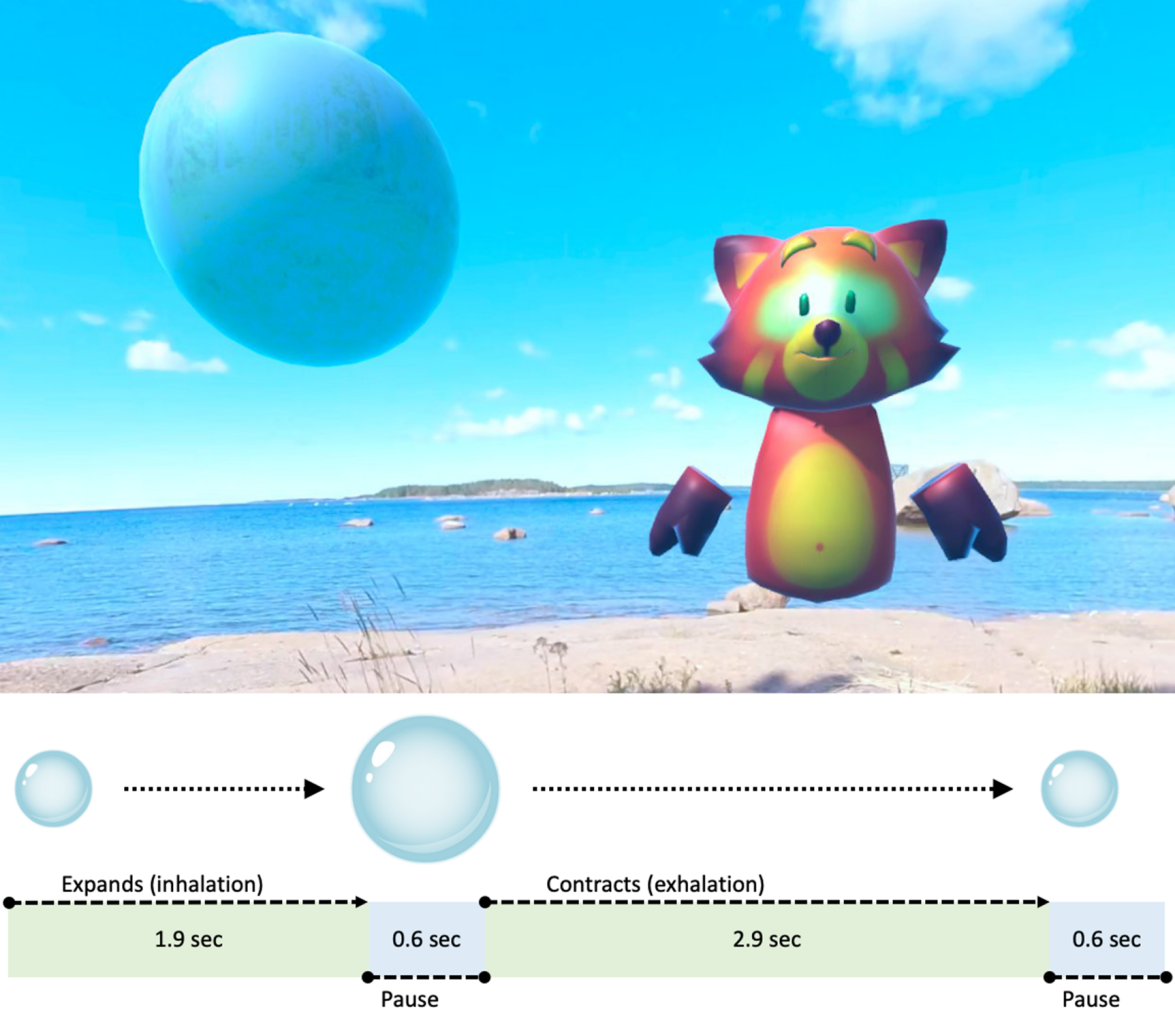
The scenery of the virtual reality relaxation application (virNE).

The study patients use a virtual reality headset while resting in a half-sitting position on a hospital bed. First, the study patients are allowed to explore the virtual natural landscape by moving their heads horizontally and vertically. After becoming comfortable with the headset, the actual 6-min guided relaxation exercise begins. The exercises take place in a virtual natural environment based on 360-degree still videos of a natural Finnish landscape (Figure 1). A gentle voice guides the patient to breathe slowly to the rhythm. To enable the patient to follow the breathing cycle, the study patient can follow a ball that is integrated into the view. This ball expands and contracts in time with the desired respiratory rate.

**Figure 2:**
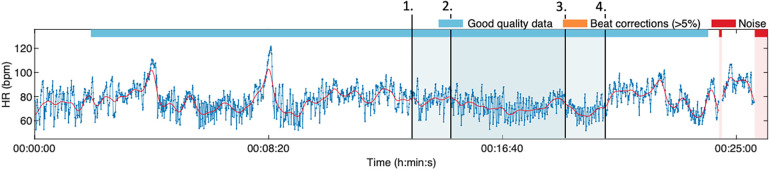
The HRV tracking during the first deep breathing session. The virtual reality headset was worn at timepoint 1. The exercise started at 2 and ended at 4. The venipuncture was performed at 3. The increased HRV can be seen during the first half of the exercise (from 2 to 3). The heart rate level elevated and HRV narrowed temporarily during the venipuncture but reverted immediately to the prior level after the procedure was finished.

The exercise manuscript includes a pause in the middle to give the study personnel a suitable point for the venipuncture. After the intravenous cannulation, the study patient will finish the exercise and is carefully aided to take off the VR headset. The response to the deep breathing exercise was monitored using a heart rate sensor belt measuring alterations in heart rate (HR) and heart rate variability (HRV) (Figure 2). Our patient experienced an immediate response to the virtual reality solution. He found the natural landscape relaxing and felt the difference when breathing slowly with the guidance. After the guided exercise and the needle procedure, he immediately commented: “I could use this in the future as well. I didn’t notice I got poked by a needle.”

**Figure 3:**
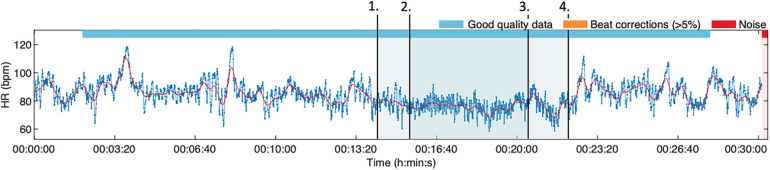
The HRV tracking during a guided relaxation training session.

Technically, the cannulation was performed easily. The next time he was cannulated, he wanted to use the VirNE-application during the procedure (Figure 3).

Since testing the VirNE-application, the patient has wanted to use it for every cannulation. The procedure itself is now effortless and can be done by a clinical nurse without any specific advance preparations. Both the patient and his parents are happy and relieved since the repetitive visits to the hospital are no longer associated with pain, fear, and nausea.

## 4 Discussion

Novel ways are needed to relieve anxiety and pain during inconvenient needle procedures. If we want to avoid patients developing long-lasting phobias concerning venipuncture and vaccination, the means of coping with the situation must be improved, thus allowing patients to modulate the fight response by themselves. Our task as health care professionals is to help youths reinforce their self-efficacy in challenging situations and thereby prepare them for responsible adulthood.

As the patient is more relaxed and confident during the needle puncture, the procedure itself can be performed technically significantly more easily due to the reduced sympathetic nervous system activation and consequently less constricted veins. Clinical studies have already shown that distraction by virtual reality environments is a safe and effective way of alleviating pain and discomfort during a procedure (13). In most of the studies, the conclusions have been based on user feedback (13, 14) instead of physiological parameters. A solid understanding of how virtual reality solutions affect the autonomic nervous system is needed, especially when using the applications for a clinical purpose. Using virtual natural environments, we have a tool that is not only feasible and cost-effective, but also easily modified and most of all immersive. By learning to use breathing as a means of reducing sympathetic nervous system activity and thereby enhancing emotional self-regulation, youths will have a psychological method they can apply to challenges in everyday life. By coupling the immersive and adjustable virtual reality solutions with educating the patient to self-regulate the fight or flight response, we can have an exquisite, novel clinical practice that is similarly suitable for self-help.

The initial cost for preparing the VirNE-application were reasonable, estimated to be about 45,000 USD. The headset costs about 500 USD. The application can be copied onto an unlimited number of new devices and the used without additional costs. This makes the system cost-effective. In our case, the VirNE-application was used in clinical trial setting with the acceptance of our local ethics committee. The use of the device in clinical setting would, however, require approval from regulatory authorities.

Finally, in our experience, when succeeding in relieving pain and anxiety, we can help the patient to cope with up-coming events as well. The spontaneous comment from the legal guardian was that the use of the application remarkably changed their family life as the nausea related to the procedure preparations and the anxiety prior to the cannulation were resolved.

## Data Availability

The raw data supporting the conclusions of this article will be made available by the authors, without undue reservation.
